# Lunch breaks as a buffer: mitigating the negative effects of road traffic noise on urban employee workplace behavior

**DOI:** 10.3389/fpubh.2025.1681862

**Published:** 2025-10-24

**Authors:** Jianglin Ke, Zhaoyue Wang, Yufei Zhang, Lidan Liu, Qiongwei Wang

**Affiliations:** ^1^School of Government, Beijing Normal University, Beijing, China; ^2^School of Public Administration and Policy, Renmin University of China, Beijing, China

**Keywords:** residential road traffic noise annoyance, mental health complaints, work withdrawal, workplace aggressive behaviors, organizational lunch break environment

## Abstract

**Introduction:**

The impact of road traffic noise annoyance on individual health is well-documented in environmental and health studies. However, less attention has been given to its negative effects on employees’ work behaviors and effective mitigation strategies.

**Methods:**

Drawing on conservation of resources theory and general strain theory, this research conducted a three-wave survey involving 816 urban employees from 304 Chinese cities.

**Results:**

The results revealed a significant positive correlation between residential road traffic noise annoyance and employees’ mental health complaints, work withdrawal, and workplace aggressive behaviors. Mental health complaints were confirmed to fully and partially mediate the relationship between noise annoyance and work withdrawal and workplace aggressive behaviors, respectively. The organizational lunch break environment negatively moderates the link between residential road traffic noise annoyance and mental health complaints.

**Discussion:**

These findings underscore the importance of addressing noise annoyance to enhance both employee welfare and organizational efficacy.

## 1 Introduction

Road traffic noise annoyance is a psychological construct defined as an individual’s cognitive and emotional evaluation of an acoustic environment influenced by vehicular noise, which may result in a feeling of partial helplessness and the need to undertake undesired actions (1). Currently, research on road traffic noise annoyance has garnered significant attention from scholars. This is due to the fact that, in contrast to objective measures of noise, noise annoyance focuses on individuals’ specific sensitivity and vulnerability to noise, which is more closely related to health outcomes (2). Among the existing studies of road traffic noise annoyance, scholars have pay attention to the measurement of road traffic noise annoyance (1, 3). The effects of acoustic parameters and non-acoustic parameters (e.g., occupation and income) on road traffic noise annoyance, and the relationship between road traffic noise annoyance and mental health issues (e.g., depression, anxiety, anger, and distraction, etc.) (4, 5). In order to gain a more comprehensive understanding of the effects of road traffic noise annoyance, some scholars have called for more research on the effects of road traffic noise annoyance on work-related attitudes and behaviors of employees (5). In the field of organizational behavior research, the issue of family–work conflict and enrichment has received widespread attention. Nevertheless, previous literature has paid limited attention to how road traffic noise annoyance manifests in the residential context, which is particularly relevant to understanding its spillover effects on employees’ family and work domains. In this study, we define residential road traffic noise annoyance (RRTNA) as a subjective psychological construct that reflects residents’ perception and evaluation of road traffic noise in their living environment. Unlike objective acoustic indicators (e.g., decibel levels), it emphasizes individuals’ subjective perception and personal appraisal of the residential acoustic environment. As an influence derived from the physical family environment, RRTNA is likely to exert diverse effects on individuals living in different family contexts, thereby shaping the dynamics of family–work conflict and gain. However, existing research offers limited insight into the impact of RRTNA on employees’ negative workplace behaviors and the ways to mitigate its adverse effects.

Previous research has established a link between noise annoyance and negative emotions such as anger and anxiety (5), which are strongly associated with employees’ adverse workplace behaviors (6). Accordingly, this study aims to investigate the impact of RRTNA on employees’ negative workplace behaviors, specifically examining both work withdrawal and workplace aggressive behaviors concurrently. The inclusion of these two outcome variables enables a comprehensive examination of how RRTNA influences such behaviors. Here, work withdrawal is considered an internalizing behavior (7), while workplace aggressive behaviors is regarded as an externalizing behavior (8), thus offering distinct perspectives on how employees respond to RRTNA. Moreover, work withdrawal can be perceived as an indirect form of aggression, which disrupts organizational productivity and service delivery while often appearing unintentional (9). In contrast, workplace aggressive behaviors involve direct forms of aggression, including physical and verbal acts (10).

Furthermore, in response to scholarly appeals for research on work withdrawal and workplace aggressive behaviors, which are prominent manifestations of negative workplace conduct, there has been a growing interest recently. This is primarily because these behaviors serve as catalysts for increased organizational costs (e.g., diminished productivity, heightened employee turnover, and reduced organizational commitment) (11, 12). Isola et al. (13) have urged researchers to delve deeper into the family-work spillover effect. Specifically, they highlight the need to examine how familial factors influence employee work withdrawal and to identify the psychological mechanisms underlying this spillover. Similarly, scholars such as Kim & James (11) have called for more investigation into the root causes of workplace aggressive behaviors, along with effective strategies to mitigate it.

The conservation of resources (COR) theory posits that adverse environmental conditions often threaten individuals’ resources or lead to resource depletion, thereby rendering them particularly vulnerable when resource access is limited (14). For instance, RRTNA may lead to actual resource loss (e.g., diminished environmental quality in residential areas) or potential resource loss (e.g., reduced individual sleep duration). These losses may prompt individuals to avert further depletion or to activate self-protective defense mechanisms (14), which often manifest as defensive withdrawal or direct aggressive behavior (15). Moreover, in accordance with stress and coping theory (16), the negative emotions associated with RRTNA may lead individuals to withdraw from work for short or extended periods as a way to protect their mental well-being. These emotions may also increase the likelihood of aggressive behavior in the workplace (17, 18).

To explore the operational mechanism, we introduced mental health complaints as a mediating factor to elucidate how RRTNA influences employees’ work withdrawal and workplace aggressive behaviors. Mental health complaints denote individuals’ subjective perception of psychological distress and the manifestation of psychological health issues (e.g., feelings of depression, anxiety, etc.) (19). According to the cognitive activation theory of stress, mental health complaints are considered as psychological stress responses resulting from stressful stimuli (20). COR theory delineates the interplay among stressors, stress responses, and individual coping behaviors (15, 21). Consistent with this theory, RRTNA acts as a stressor and induces psychological stress responses manifested as mental health complaints. These responses, in turn, lead employees to adopt defensive strategies to prevent resource loss, which often culminate in defensive withdrawal or aggressive behavior. Furthermore, according to the general strain theory (22), RRTNA, as a negative stressor, can exacerbate individuals’ mental health complaints, thereby heightening the likelihood of work withdrawal or workplace aggressive behaviors.

Furthermore, the organizational environment can subtly influence employees. We examined a potential boundary condition and investigated how the organizational lunch break environment moderates this relationship. Unlike Western cultures, Chinese individuals typically prefer taking naps during lunch breaks. This preference may stem from cultural differences in eating habits. Chinese employees generally consume larger meals at lunchtime than their Western counterparts (23). Such dietary patterns may contribute to postprandial reactive hypoglycemia, lethargy, and drowsiness. Lunch breaks are therefore particularly important for Chinese employees in restoring energy and reducing daily work-related stress (24, 25). Consistent with COR theory (15), a supportive organizational lunch break environment can serve as a positive environmental resource that alleviates the adverse effects of RRTNA and moderates its association with somatic mental health complaints.

To address the existing research gap, this study investigates the mediating role of mental health complaints between RRTNA and work withdrawal and workplace aggressive behaviors, as well as the moderating influence of the organizational lunch break environment, focusing on Chinese urban employees. This study contributes to the literature in several ways. First, it broadens the scope from environmental and health domains to the management field by investigating how RRTNA influences employee mental health complaints, work withdrawal, and workplace aggressive behaviors. This responds to calls by scholars such as Manohare et al. (5) for further examination of how road traffic noise annoyance affects the work behaviors of adjacent populations. Second, it introduces a negative mental health perspective and proposes a mediation mechanism involving mental health complaints, offering new insights into the mechanisms of road traffic noise annoyance effects. Third, it identifies and verifies an effective strategy to mitigate the adverse effects of RRTNA on employees’ mental health complaints, work withdrawal, and workplace aggressive behaviors—the moderating role of the organizational lunch break environment. Fourth, this study makes significant theoretical contributions by extending both the COR theory and the general strain theory. For COR theory, we broaden its application by identifying RRTNA as a novel antecedent that depletes personal resources from the home domain, which subsequently spills over to the work domain. More importantly, we introduce and validate the organizational lunch break environment as a critical contextual resource that can buffer this resource loss process. This delineates a more complete cross-domain resource gain and loss spiral, enriching the COR framework. For general strain theory, we respond to the call for identifying specific mechanisms linking strain to outcomes by introducing mental health complaints as a pivotal mediating mechanism. Our findings indicate that mental health complaints fully mediate the link to work withdrawal and partially mediate the link to aggression. This provides a more nuanced understanding of the psychological pathways through which environmental strains translate into divergent workplace behaviors, thereby refining the general strain theory. Practically, this study suggests ways for organizations to provide employees with appropriate resources and support to alleviate the negative impacts of RRTNA.

## 2 Literature review and research hypothesis

### 2.1 Residential road traffic noise annoyance and work withdrawal, workplace aggressive behaviors

Consistent with Schreckenberg et al. (1), we believe that RRTNA comprises three dimensions. First, individuals’ experiences of disturbance from residential road traffic noise. Second, emotional and attitudinal responses to this noise. Third, the perceived inability to cope with it. Previous studies have linked RRTNA to negative reactions such as anger, anxiety, and distraction (5), which, in turn, correlate strongly with work withdrawal and workplace aggressive behaviors (26, 27). Thus, we hypothesize that RRTNA positively influences employees’ tendencies toward work withdrawal and workplace aggressive behaviors.

Examining work withdrawal and workplace aggressive behaviors as outcome variables of RRTNA allows for a comprehensive understanding of the impact of RRTNA on both implicit and explicit negative workplace behaviors. Work withdrawal is often regarded as an implicit behavior, meaning that it is difficult to observe externally (28). It is defined as employees’ avoidance of their work roles and tasks while maintaining their organizational role and functional ties. In this process, employees reduce the time and energy devoted to work, for example by developing thoughts of not attending work (12). In contrast, direct aggression is outwardly visible, involving disruptive behaviors that clearly violate social norms and may harm others (29). Workplace aggressive behavior is defined as the deliberate actions of organizational members intended to harm their organization or other members within it (30). Research indicates that workplace aggression is also relatively common in some Chinese organizations (31). Compared to Western countries, Chinese employees tend to exhibit more subtle and indirect forms of aggression, reflecting the long-standing cultural value of maintaining harmony.

COR theory explains how people behave in stressful environments by suggesting that environmental conditions often threaten or deplete individuals’ resources (14). When individuals lack the ability to acquire resources, they become particularly vulnerable and tend to protect their remaining resources or activate self-protective defense mechanisms (14). Noise annoyance signifies a lack of coping resources against environmental health threats (32), prompting individuals to safeguard their resources or activate self-protective mechanisms accordingly. Nauman et al. (33) argue that work withdrawal stems from employees’ defensive efforts to protect their remaining resources. Similarly, Halbesleben et al. (34) posit that individuals who activate self-protection mechanisms are more prone to exhibiting aggressive behaviors.

Moreover, according to stress and coping theory, individuals continually appraise environmental stimuli, and this evaluative process elicits emotional responses (35). When a stimulus is perceived as threatening or harmful, individuals engage in coping efforts to address the stressor or alleviate negative emotions (35). For instance, Lee et al. (36) demonstrated that low life satisfaction, reflecting negative evaluations of one’s living environment, can lead to maladaptive coping behaviors such as drug use or aggression. RRTNA reflects individuals’ negative appraisal of their residential environment and is often accompanied by emotions such as helplessness and tension (1). It is typically driven by both the objective environmental noise levels, measured in decibels, and individual sensitivity to noise (37). Specifically, when exposed to the same objective environmental noise levels in decibels, individuals with higher noise sensitivity are likely to report higher levels of RRTNA. In the workplace, individuals experiencing negative emotions are inclined to mitigate their tension by either temporarily withdrawing from work or engaging in aggressive behaviors, which may culminate in overt manifestations of aggression (17, 18).

Based on this, the following hypothesis is proposed:

**H1**: RRTNA has a positive effect on work withdrawal and workplace aggressive behaviors.

### 2.2 Residential road traffic noise annoyance and mental health complaints

The environment’s influence on an individual’s mental well-being has been noted (38, 39), with research indicating a close link between both objectively measured environmental factors and individuals’ subjective perceptions of their surroundings and mental health (40). Physical parameters can provide fairly objective measurements of noise exposure, while inquiries regarding noise interference can assess individuals’ perceptions and evaluations of sound (41). Given that not all individuals interpret loud sounds as noise, noise annoyance serves as a particularly relevant indicator for exploring the connection between noise disturbance and health outcomes (40).

Indregard et al. (42) argued that health risks perceived from potential environmental threats can influence individuals’ perceptions of their health, leading to the occurrence of mental health complaints. This perspective is supported by COR theory. COR theory defines psychological stress as a response to an environment involving the potential or actual loss of resources, and notes that both perceived and actual losses can trigger psychological stress responses, such as anxiety (14). Applying this logic, RRTNA indicates a decline in the residential physical environment’s quality, potentially leading to a depletion of energy resources, such as reduced sleep duration. Consequently, this actual and potential loss of physical and energy resources may trigger individuals to experience psychological stress responses, with mental health complaints regarded as manifestations of such stress responses (20).

According to the cognitive activation theory of stress, stressors elicit stress responses. When an individual perceives a discrepancy between the actual and desired values, it triggers an alarm reaction characterized by heightened stress or physiological arousal (43). This alarm reaction is not indicative of a conventional mental illness but rather of what is termed a subjective mental health complaint (20). Applying this theory, noise annoyance signifies a disparity between the actual and desired living environments, thereby resulting in individual mental health complaints.

Based on this, the following hypothesis is proposed:

**H2**: RRTNA has a positive effect on mental health complaints.

### 2.3 Mental health complaints and work withdrawal, workplace aggressive behaviors

According to COR theory, individuals prioritize the protection and acquisition of resources, including energy resources (e.g., knowledge and time), which facilitate the acquisition of other vital resources (e.g., social support, financial assets, and emotional stability) (14). Building upon this framework, Fleuren et al. (44) emphasize the significance of mental health status as a vital energy resource enabling individuals to pursue improved quality of life and well-being.

LeBreton et al. (45) demonstrated that individuals with poor mental health, such as psychological depression, are prone to engaging in withdrawal behaviors. Similarly, Balducci et al. (46) found that poor mental health (e.g., anxiety and depression) increases the likelihood of individuals displaying workplace aggressive behaviors compared to short-term negative affect. COR theory offers an explanation for the association between mental health complaints and both work withdrawal and workplace aggressive behaviors. Elevated levels of mental health complaints indicate depleted energy resources. According to COR theory, when individuals’ resources are depleted, they become more cautious in investing future resources and are more inclined to adopt a defensive stance in utilizing their remaining resources, thereby resulting in work withdrawal and workplace aggressive behaviors (21).

Based on this we propose the following hypothesis:

**H3**: Mental health complaints have a positive effect on work withdrawal and workplace aggressive behaviors.

### 2.4 The mediating role of mental health complaints

COR theory suggests that when individuals experience a loss of resources, they respond with stress, prompting defensive actions to prevent further loss (15, 21). This framework explains the link between stressors, stress responses, and individual coping behaviors. Accordingly, RRTNA induces psychological stress, leading employees to adopt defensive measures to avert resource loss, which may manifest as either defensive withdrawal or aggressive behavior.

Moreover, according to general strain theory, negative stimuli can heighten psychological distress (e.g., anger or frustration), potentially leading individuals to engage in risky behaviors like aggression (36). Building on this, RRTNA, as a negative stressor, may exacerbate individuals’ mental health complaints, thereby increasing the likelihood of work withdrawal or workplace aggressive behaviors.

Based on this, the following hypothesis is proposed:

**H4**: Mental health complaints mediate the relationship between RRTNA and work withdrawal and the relationship between RRTNA and workplace aggressive behaviors.

### 2.5 The moderating role of organizational lunch break environment

In organizational contexts, lunch break is defined as ceasing work at midday to rest and unwind (47). Venz et al. (48) introduced the concept of person-break fit within the framework of person-environment fit, highlighting its role in helping employees preserve resources and significantly impacting their afternoon work performance. Person-break fit posits that when an individual’s break aligns with their needs, such as relaxation, it enhances their satisfaction and contributes to their overall role fulfillment during afternoon work. This fit is influenced by various factors of the break, including the break experience and environment, which affect the individual’s satisfaction with their needs. For instance, a noisy or unsatisfactory resting environment can impede relaxation needs fulfillment. Building on this notion, this study defines the organizational lunch break environment as the resources provided by the organization to support employees’ lunch break needs within the workplace. These resources include designated lunch break areas and quiet environments conducive to rest.

A higher level of organizational lunch break environment is characterized by ample resources provided for employees’ lunch breaks. Such resources help meet employees’ needs, thereby promoting person-break fit and enabling them to resist resource loss (48). According to COR theory, infusing and enhancing resources is crucial in mitigating tension and stress responses resulting from stressors (15). Therefore, a favorable organizational lunch break environment can moderate the impact of RRTNA on mental health complaints. When organizations provide employees with a supportive lunch break environment, they gain more opportunities and resources to restore energy and relieve stress during breaks. Even when exposed to road traffic noise annoyance in their residential environment, the likelihood of experiencing mental health complaints is reduced. Furthermore, the cognitive activation theory of stress suggests that both environmental factors and individual resources can moderate the impact of stressors on stress responses (49).

Based on this, the following hypothesis is proposed:

**H5**: Organizational lunch break environment negatively moderates the relationship between RRTNA and mental health complaints.

### 2.6 Moderated mediation model

Drawing on H4 and H5, this study proposes a moderated mediation model. Specifically, it suggests that mental health complaints mediate the relationship between RRTNA and work withdrawal, as well as workplace aggressive behaviors. However, the strength of this mediating effect hinges on the organizational lunch break environment. In contexts where the lunch break environment is more supportive, employees are better equipped to mitigate the stress induced by RRTNA through the lunch break. This, in turn, reduces individual mental health complaints, subsequently diminishing work withdrawal and workplace aggressive behaviors.

Based on this, the following hypotheses are proposed:

**H6a**: Organizational lunch break environment negatively moderates the indirect relationship between RRTNA and work withdrawal through mental health complaints.

**H6b**: Organizational lunch break environment negatively moderates the indirect relationship between RRTNA and workplace aggressive behaviors through mental health complaints.

**Figure 1:**
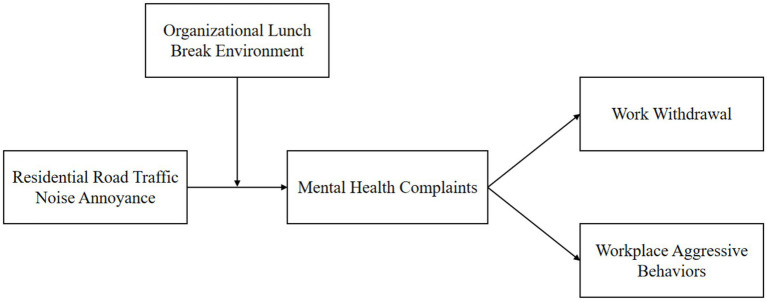
Research model.

Based on the above analysis, the research model established in this study is shown in Figure 1.

## 3 Research method

### 3.1 Sample source

This study utilized WENJUANXING, a crowd-sourcing platform in China similar to Amazon Mechanical Turk, to distribute and collect electronic questionnaires from office-based working employees in the city. The survey was available to participants starting from November 2024. To mitigate common method bias, a three-wave survey with monthly data collection was employed. Each participant’s questionnaire responses were tracked using a non-identifiable matching code, and distribution of the questionnaire was monitored at specific intervals. All responses were collected anonymously, with participants being assured that the data would remain confidential and be used exclusively for academic research purposes. For data collection, RRTNA and organizational lunch break environment were measured at time one, mental health complaints were measured at time two, and work withdrawal and workplace aggressive behaviors were measured at time three.

A total of 1,203 questionnaires were collected during the three-wave survey. Throughout the data collection process, 385 questionnaires were deemed unqualified due to factors such as consistent responses or failed lie detector questions, resulting in 816 valid questionnaires remaining, representing an effective rate of 68%. Although convenience sampling was employed, the sample comprised participants from nearly all provinces across mainland China, with sample proportions closely mirroring the population distribution of each province. Thus, the sample data exhibits good coverage and representativeness. The sample structure is as follows. Gender: 38.5% male and 61.5% female. Age: 36.2% 30 and under, 32.5% 31–40, 23.4% 41–50, 7.9% 50 and over. Work age: 7.6% less than 1 year, 21.0% 1–5 years, 29.3% 5–10 years, 16.1% 10–20 years, 26.0% 20 years and over. Average working hours per day: 15.8% less than 7 h, 37.8% 7–8 h, 30.2% 8–9 h, 10.2% 9–10 h, and 6.0% more than 10 h.

### 3.2 Measurement tools

In this study, well-established scales were used, and some of the scales were partially revised to fit the actual context. Each scale was scored on a five-point Likert scale (“1 = strongly disagree, 2 = disagree, 3 = neutral, 4 = agree, 5 = strongly agree”). The scale has good reliability, with Cronbach’s Alpha internal consistency coefficients reaching above 0.80.

#### 3.2.1 Residential road traffic noise annoyance

We used the road traffic noise annoyance scale developed by Schreckenberg et al. (1), with seven items. The items contained in this scale primarily target the subjective perception of noise among participants within their residential setting. Specifically, the scale aims to evaluate subjects’ subjective understanding and awareness of noise intensity and characteristics within their living environment. Unlike directly addressing the physical attributes of noise, such as decibel levels, this assessment predominantly focuses on how individuals perceive and interpret these auditory stimuli. The scale encompasses inquiries pertaining to subjects’ perceptions regarding the frequency, duration, extent of impact, and level of discomfort or disruption caused by noise. The results of the exploratory factor analysis of the original scale included “I know that I can protect myself quite well against road traffic noise” and “If it is too loud outside, I simply close the window, and then I am no longer disturbed.” The factor loadings of these two questions were too low, 0.529 and 0.565 respectively, and the factor loadings of these two questions in this survey were 0.180 and 0.092, which were less than 0.5, so these two questions were deleted and the final scale had 5 items. Cronbach’s Alpha = 0.90, χ^2^/*df* = 1.28, RMSEA = 0.031, CFI = 0.999, TLI = 0.997.

#### 3.2.2 Mental health complaints

We used a five-item scale adopted from Berwick et al. (50). Sample items are “I’ve been a very anxious person” and “I felt calm and peaceful.” Cronbach’s Alpha = 0.85, χ^2^/*df* = 1.19, RMSEA = 0.025, CFI = 0.999, TLI = 0.997.

#### 3.2.3 Work withdrawal

We used the scale developed by Lehman and Simpson (51) and we modified it with six items. Sample items are “Thought of being absent” and “Chat with co-workers about non-work topics.” Cronbach’s Alpha = 0.84, χ^2^/*df* = 1.73, RMSEA = 0.049, CFI = 0.989, TLI = 0.982.

#### 3.2.4 Workplace aggressive behaviors

We used a modified Chinese context-based workplace aggressive behaviors scale by Xu & Tian (52) with seven question items. Sample items are “I damaged property belonging to my employer” and “I said or did something to purposely hurt someone at work.” Cronbach’s alpha = 0.93, χ^2^/*df* = 2.76, RMSEA = 0.076, CFI = 0.987, TLI = 0.978.

#### 3.2.5 Organizational lunch break environment

The study was adapted based on the service environment scale developed by Taylor & DiPietro (53) and combined with the actual situation of Chinese people organizing lunch breaks. The final scale was a single dimension with a total of 5 items, and the cumulative explained variance was 69.717%. The loading of each factor was above 0.5 (see Table 1), and the scale had good structural validity. Cronbach’s Alpha = 0.89, χ^2^/*df* = 1.57, RMSEA = 0.043, CFI = 0.996, TLI = 0.990.

**Table 1:** Exploratory factor analysis of organizational lunch break environment.

Question	Factor loading
My organization provides the opportunity and resources to ensure my lunch break.	0.645
There are places within the organization for employees to take lunch breaks.	0.775
A quiet lunch break environment is provided.	0.778
A lunch break environment with appropriate brightness is provided.	0.700
Appropriate temperatures for employees during lunch breaks by using air conditioners or opening windows for ventilation, etc. are provided.	0.753

#### 3.2.6 Control variable

Following previous research (54, 55) and in conjunction with our study, we controlled for employees’ demographic characteristics, including gender, age, work age, average working hours per day.

## 4 Data analysis and results

### 4.1 Confirmatory factor analysis

According to Schmidt & Finan (56), the normality of the test model residuals is sufficient to satisfy the prerequisites for hypothesis testing. Upon examination, the assumption of normality has been met. Mplus 8.3 software was utilized to assess the discriminant validity of the entire model. To confirm the discriminant validity of the key variables “RRTNA,” “mental health complaints,” “work withdrawal,” “workplace aggressive behaviors,” and “organizational lunch break environment,” a confirmatory factor analysis was conducted to compare various models including the five-factor model, four-factor model, three-factor model, two-factor model, and one-factor model. The findings presented in Table 2 indicate that the five-factor model demonstrates the most favorable fit and significantly outperforms the other four models. This suggests that the five-factor model exhibits strong discriminant validity and offers a superior representation of the factor structure within the measurement model.

**Table 2:** Confirmatory factor analysis.

Model	χ^2^	df	χ^2^/*df*	RMSEA	CFI	TLI
Five-factor	760.406	310	2.453	0.069	0.920	0.903
Four-factor	1912.494	344	5.560	0.122	0.722	0.695
Three-factor	2648.839	347	7.634	0.148	0.593	0.556
Two-factor	3198.763	349	9.166	0.164	0.496	0.454
One-factor	3390.473	350	9.687	0.169	0.462	0.419

Five-factor model: Including RRTNA, mental health complaints, work withdrawal, workplace aggressive behaviors, and organizational lunch break environment. Four-factor model: On the basis of the five-factor model, the organizational lunch break environment and RRTNA were combined into one factor. Three-factor model: Based on the five-factor model, mental health complaints, organizational lunch break environment, and RRTNA were combined into one factor. Two-factor model: Based on the five-factor model, workplace aggressive behaviors, mental health complaints, organizational lunch break environment, and RRTNA were combined into one factor. One-factor model: The five variables were combined into one factor.

### 4.2 Common method biases

To mitigate common method biases, Harman’s single-factor test was employed to assess the extent of such biases among the variables. The findings revealed that a single factor accounted for only 31.424% of the variance in the subjective variables, well below the 50% threshold suggested by Harrison et al. (57). Therefore, the presence of significant common method biases was deemed unlikely.

### 4.3 Descriptive statistics and correlation among study variables

Mean values, standard deviations, and correlation coefficients of the variables were computed using SPSS software for statistical analysis, with the results displayed in Table 3. Significant positive correlations were observed between RRTNA and mental health complaints, work withdrawal, and workplace aggressive behaviors (*r* = 0.23, *p* < 0.01; *r* = 0.26, *p* < 0.01; *r* = 0.41, *p* < 0.01). Additionally, mental health complaints exhibited significant positive correlations with work withdrawal and workplace aggressive behaviors (*r* = 0.51, *p* < 0.01; *r* = 0.42, *p* < 0.01).

**Table 3:** Descriptive statistics and correlations results.

Variables	M	SD	1	2	3	4	5	6	7	8	9
1. Gender	0.62	0.49	-								
2. Age	2.03	0.96	−0.18**	-							
3. Work age	3.32	1.27	−0.16**	0.85**	-						
4. Average working hours per day	2.53	1.06	−0.11*	0.04	0.12*	-					
5. Residential Road Traffic Noise Annoyance	2.91	0.98	−0.07	0.14**	−0.14*	−0.03	-				
6. Mental Health Complaints	2.71	0.82	0.10	−0.22**	−0.23**	0.05*	0.19**	-			
7. Work Withdrawal	2.86	0.76	0.04	−0.34**	−0.35**	−0.18**	0.22**	0.50**	-		
8. Workplace Aggressive Behaviors	2.07	0.92	0.01	−0.26**	−0.23**	−0.04	0.37**	0.39**	0.67**	-	
9. Organizational Lunch Break Environment	3.20	0.88	−0.04	−0.05	−0.09	−0.1	0.50**	−0.39**	−0.18**	0.09	-

*N* = 816, *p < 0.05, **p < 0.01.

### 4.4 Hypothesis testing

#### 4.4.1 Test of main effect and mediating effect

In this study, regression analysis method was used to test the main and mediating effects, and the results are shown in Table 4. The results show that RRTNA has a significant positive effect on mental health complaints, work withdrawal, and workplace aggressive behaviors (β = 0.17, *p* < 0.01; β = 0.17, *p* < 0.01; β = 0.34, *p* < 0.01). Mental health complaints have a significant positive effect on work withdrawal and workplace aggressive behaviors (β = 0.41, *p* < 0.01; β = 0.34, *p* < 0.01). Consequently, Hypotheses H1, H2 and H3 are verified. M5 shows that after adding the mediating variable of mental health complaints, the positive effect of RRTNA on work withdrawal was not significant (β = 0.07, *p* = 0.054), indicating that mental health complaints completely mediated the relationship between RRTNA and work withdrawal, using SPSS analysis software’s PROCESS plug-in yielded an indirect effect value of 0.06, CI = [0.017, 0.108], with a confidence interval that excluding 0, so the indirect effect was significant. Besides, M8 shows that after putting in the mediating variable of mental health complaints, there is a significant positive effect of RRTNA on workplace aggressive behaviors (β = 0.27, *p* = <0.01), indicating that mental health complaints partially mediated the relationship between RRTNA and workplace aggressive behaviors, using SPSS analysis software’s PROCESS plug-in yielded an indirect effect value of 0.05, CI = [0.015, 0.086], with a confidence interval that excluding 0, so the indirect effect is significant. Thus, Hypothesis H4 is verified.

**Table 4:** Test results of main effect and mediating effect.

Variables	Mental Health Complaints		Work Withdrawal			Workplace Aggressive Behaviors		
	M1	M2	M3	M4	M5	M6	M7	M8
Gender	0.07	0.08	−0.04	−0.02	−0.09	−0.04	−0.01	−0.06
Age	−0.05	−0.04	−0.21*	−0.20	−0.14*	−0.23*	−0.19	−0.18
Work age	−0.19	−0.17	−0.15	−0.14	−0.04	−0.04	−0.02	0.03
Average working hours per day	0.08	0.09	−0.15**	−0.15***	−0.13**	−0.03	−0.02	−0.04
Residential Road Traffic Noise Annoyance		0.17**		0.17**	0.07		0.34**	0.27**
Mental Health Complaints					0.41**			0.34**
F	5.17**	6.12**	13.29**	12.96**	27.87**	5.49**	13.35**	18.09**
Δ*R*^2^		0.02		0.03	0.18		0.11	0.09
*R* ^2^	0.07	0.09	0.15	0.18	0.36	0.07	0.18	0.27

*p < 0.05, **p < 0.01.

#### 4.4.2 Test of moderating effect

Table 5 presents findings on the moderating influence of the organizational lunch break environment. M2 reveals a statistically significant positive association between RRTNA and mental health complaints (β = 0.49, *p* < 0.01), while M3 demonstrates a significant negative interaction effect between the organizational lunch break environment and RRTNA on mental health complaints (β = −0.12, *p* < 0.01). This suggests that the organizational lunch break environment acts as a negative moderator in the relationship between RRTNA and mental health complaints, thereby confirming hypothesis H5.

**Table 5:** Test results of moderating effects.

Variables	Mental Health Complaints		
	M1	M2	M3
Gender	0.07	0.08	0.15
Age	−0.05	0.04	0.04
Work age	−0.19	−0.25**	−0.15**
Average working hours per day	0.08	0.03	0.01
Residential Road Traffic Noise Annoyance		0.49**	0.82**
Moderating Effect			
Organizational Lunch Break Environment		−0.65**	−0.29*
Residential Road Traffic Noise Annoyance × Organizational Lunch Break Environment			−0.12**
*F*	5.17**	33.26**	30.56**
Δ*R*^2^		0.33	0.02
*R* ^2^	0.07	0.40	0.42

*p < 0.05, **p < 0.01.

**Figure 2:**
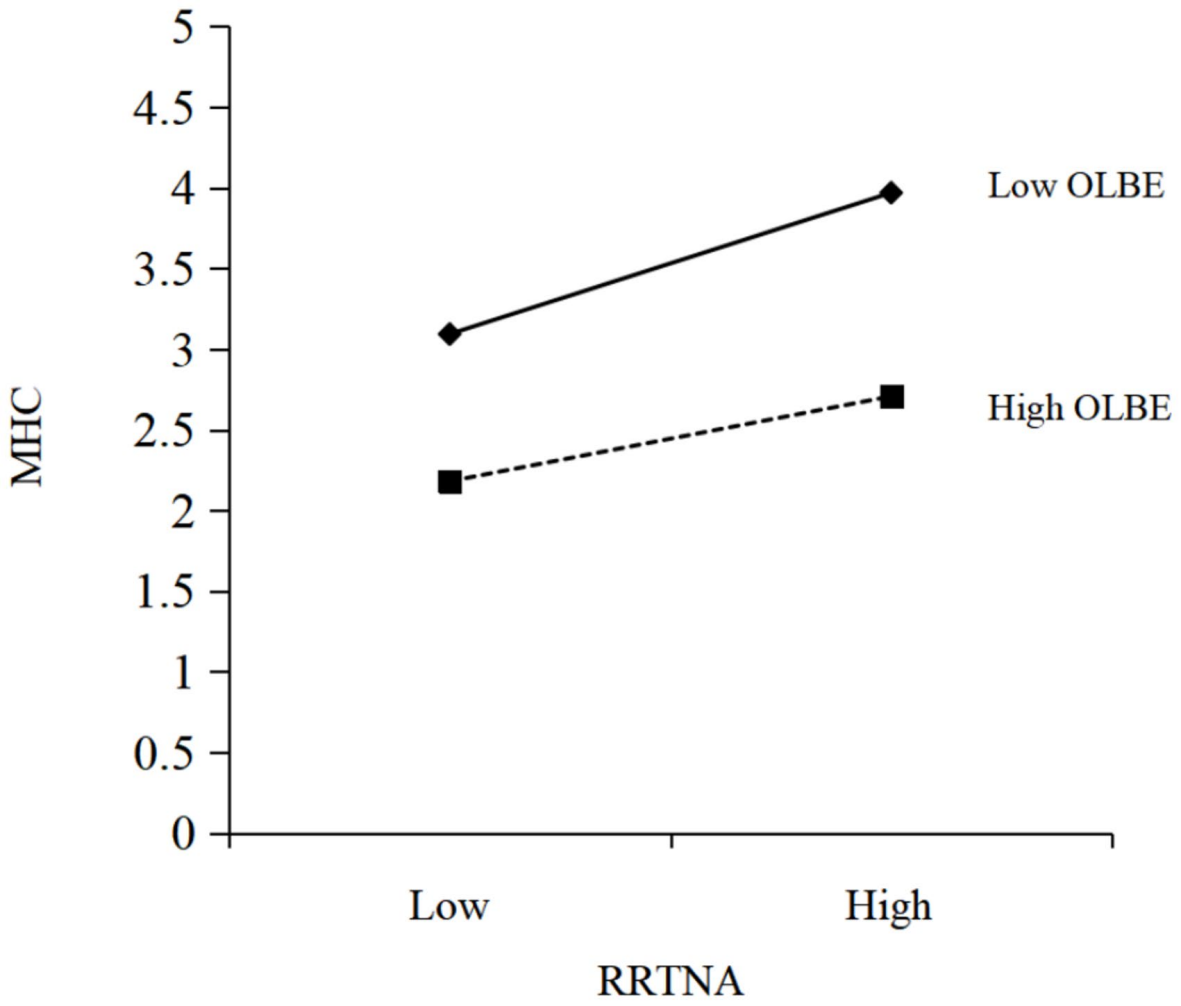
Moderating effect of organizational lunch break environment. RRTNA, Residential Road Traffic Noise Annoyance; MHC, Mental Health Complaint; OLBE, Organizational Lunch Break Environment.

To further confirm whether the moderating effect of organizational lunch break environment in the relationship between RRTNA and mental health complaints is as expected, we refer to Aiken et al. (58), add the value of organizational lunch break environment plus or minus one standard deviation to the regression model, and plot it to obtain Figure 2. The higher the score of the organizational lunch break environment, the richer resources the organization provides for employees to take their lunch break in the organization. As can be seen in Figure 2, the positive association between RRTNA and mental health complaints is weaker when the level of organizational lunch break environment is higher. This shows that the resulting moderating effect results are in line with expectations.

#### 4.4.3 Test of moderated mediation effect

Hypotheses H6a and H6b posit that the organizational lunch break environment moderates the indirect effect of RRTNA on work withdrawal and workplace aggressive behaviors through mental health complaints. Following Edwards and Lambert (59), we incorporated the organizational lunch break environment value plus or minus one standard deviation into the regression model. Table 6 shows the comparison results of the differences in the moderated mediation models. The indirect effect of RRTNA on work withdrawal through mental health complaints is 0.13 versus 0.22 at high and low levels of organizational lunch break environment, respectively, with a 95% CI of [−0.145, −0.023] (excluding 0) for the difference (−0.09) reaching a significant level. This suggests that the impact of RRTNA on work withdrawal through mental health complaints is notably greater when the quality of the organizational lunch break environment is low compared to when it is high, thereby confirming hypothesis H6a. Similarly, the indirect effect of RRTNA on workplace aggressive behaviors through mental health complaints is 0.11 versus 0.18 at high and low levels of the organizational lunch break environment, respectively. The 95% confidence interval for the difference (−0.07) does not include zero, indicating a significant difference. This implies that the impact of RRTNA on workplace aggressive behaviors through mental health complaints is significantly stronger when the organizational lunch break environment is low compared to when it is high, supporting hypothesis H6b.

**Table 6:** Test results of moderated mediation effect.

Mediation path	Organizational lunch break environment	indirect effect	S.E.	95% CI
Residential Road Traffic Noise Annoyance → Mental Health Complaints → Work Withdrawal	High	0.13	0.028	[0.084, 0.192]
	Low	0.22	0.036	[0.152, 0.291]
	Diff.	−0.09	0.031	[−0.145, −0.023]
Residential Road Traffic Noise Annoyance → Mental Health Complaints → Workplace Aggressive Behaviors	High	0.11	0.025	[0.066, 0.163]
	Low	0.18	0.034	[0.120, 0.250]
	Diff.	−0.07	0.026	[−0.124, −0.021]

CI, Confidence Interval.

## 5 Research findings and discussion

Based on the hypotheses proposed and tested earlier, all hypotheses put forward in this study are confirmed. This study focuses on urban employees in China to develop and test a model examining the impact of RRTNA on their work withdrawal and workplace aggressive behaviors. The findings extend the scope of research on traffic noise annoyance by demonstrating, from the perspective of negative mental health, its positive effects on both work withdrawal and aggressive behaviors at work, as well as the underlying mechanisms involved. The results are then discussed, followed by practical managerial implications and suggestions for future research.

### 5.1 Discussion of research findings

First, this study demonstrates that RRTNA affects both employees’ mental health complaints and work performance, thus providing theoretical and empirical support for examining its impact on neighborhood residents. The results indicate a significant positive effect of RRTNA on employees’ mental health complaints, work withdrawal, and workplace aggressive behaviors (see Table 3)and the main and mediated effects analyses (Table 4) further confirm these findings. While previous research on road traffic noise annoyance has primarily focused on environmental and health domains, its relationship with workplace behaviors remains underexplored. By linking road traffic noise annoyance to the management field, this study responds to scholars’ calls for further research on nearby residents effects (5) and provides deeper insights into how the home environment shapes employees’ work. Theoretically, this study extends COR theory by demonstrating that resource depletion is not only confined to traditional workplace stressors but can also stem from the physical environment of the residential domain. By identifying RRTNA as a critical antecedent of work behaviors, this study broadens the scope of COR theory to a more holistic, cross-domain perspective on resource loss and gain spirals.

Second, this study demonstrates that mental health complaints mediate the relationship between RRTNA and work withdrawal and workplace aggressive behaviors, offering a new theoretical perspective on the behavioral impact of RRTNA. Previous studies on the effects of noise on individual behavior have examined mediators such as noise perception and behavioral intention (60, 61). Although prior research has shown that continuous road traffic noise annoyance affects individuals’ psychological well-being (4) and established a strong link between employees’ psychological health and work withdrawal or workplace aggressive behaviors (62, 63), no study has specifically investigated the mediating role of mental health complaints in the relationship between RRTNA and work-related behaviors.

Consequently, this study introduces mental health complaints as a novel mediator. Drawing upon COR theory, it suggests that elevated levels of RRTNA can trigger negative emotions, leading individuals to perceive a decline in their mental well-being. To compensate for depleted energy resources and protect themselves, individuals may withdraw from work or exhibit aggressive behaviors. By applying COR theory, this research aims to illuminate the underlying mechanisms linking RRTNA to employee work withdrawal and workplace aggressive behaviors. In doing so, it integrates RRTNA into organizational management research and extends road traffic noise annoyance studies by highlighting its impact on employees’ behavioral responses. The proposed mediating mechanism also contributes to theoretical refinement. Within COR theory, it clarifies the resource being depleted—psychological well-being and mental energy—at the core of the loss spiral. For general strain theory, it advances beyond the broad notion of negative affect by identifying a distinct psychological state that explains the pathway between environmental strain and behavior. Thus, the findings extend general strain theory by uncovering a pivotal mechanism through which strain manifests in the workplace.

Third, given the cultural context in which Chinese employees commonly take lunch breaks, this study proposes an effective approach to mitigating the impact of RRTNA on mental health. It shows that an organizational lunch break environment significantly moderates the relationship between RRTNA and mental health complaints. While previous studies have primarily focused on the health benefits of energy recovery during lunch breaks (64), they have paid limited attention to the moderating role of the organizational lunch break environment in relation to negative mental health outcomes. According to COR theory, a higher-quality lunch break environment provides employees with additional resources to alleviate the stress responses caused by residential road traffic noise, thereby reducing their mental health complaints. This finding makes a novel theoretical contribution to COR theory by conceptualizing and empirically validating the organizational lunch break environment as a valuable contextual resource. While COR theory has traditionally focused on personal resources and social resources (e.g., self-efficacy, emotional support) (65), this study highlights that recovery-supportive physical environments provided by the organization can effectively buffer resource loss initiated by non-work stressors. This shifts the theoretical discussion from individual resource management to the organization’s active role in providing contextual resources for recovery, thereby enriching the COR framework with a more organizational and design-oriented perspective.

Fourth, this study identifies an effective approach to mitigating the effects of residential road traffic noise on work, constructed a moderated mediation model, and verified the effects of the organizational lunch break environment on “RRTNA → mental health complaints → work withdrawal” and “RRTNA → mental health complaints → workplace aggressive behaviors.” Taken together, this moderated mediation model integrates the principles of COR theory and general strain theory into a unified framework. It explains how the interaction between RRTNA and the organizational lunch break environment influences employees’ work withdrawal and workplace aggressive behaviors through mental health complaints. In doing so, it clarifies both intrinsic motivational processes and extrinsic contextual mechanisms. It further demonstrates how an environmental strain elicits negative psychological states, which deplete personal resources, leading to negative outcomes, and how organizational-level resources can mitigate this detrimental process. This integration provides a more comprehensive theoretical lens for understanding the complex interplay between non-work stressors, individual psychological states, organizational contexts, and workplace behaviors.

### 5.2 Management practice implications

This study advances understanding of the effects of RRTNA on employees’ mental health and work performance and offers the following managerial implications. Beyond the organizational boundary, the findings hint at the broader societal relevance of residential noise as a public health concern, with possible spillover into the economic domain through workplace performance. Organizational actions are thus not only beneficial for firm-level outcomes but may also represent a critical part of the response to a common urban environmental stressor.

First, as indicated by the study findings, RRTNA positively influences employees’ work withdrawal and workplace aggressive behaviors, consequently affecting both individual and organizational performance. Hence, organizations are encouraged to implement measures aimed at encouraging and supporting employees in mitigating the effects of residential road traffic noise. For instance, organizations can provide tangible support, such as offering a yearly stipend or subsidy for employees to purchase high-quality noise-canceling headphones or earplugs, or sponsoring the installation of sound-proofing windows for employees living in notoriously noisy areas. Moreover, the feasibility of such interventions differs across organizations. Large corporations with greater resources may provide direct financial subsidies or housing-related support, while middle and small-sized enterprises could adopt more cost-effective solutions, such as collective purchasing of noise-canceling devices or community-level soundproofing initiatives. This differentiation ensures that recommendations remain realistic across organizations of varying sizes.

Second, this study reveals that mental health complaints mediate the relationship between RRTNA and employees’ work withdrawal and workplace aggressive behaviors. This finding underscores the need for organizational managers to prioritize employees’ mental health as a means to foster engagement and mitigate withdrawal and aggression. Targeted initiatives may include regular health assessments and training programs (e.g., stress management or mindfulness courses) aimed at promoting psychological well-being (66). Managers can also establish fair performance appraisal criteria to reduce undue pressure, identify workplace factors harmful to mental health, and implement comprehensive wellness guidelines (67). In physically demanding sectors with rigid schedules (e.g., manufacturing, logistics), interventions may emphasize fatigue recovery and stress alleviation. By contrast, in knowledge-intensive or digital sectors (e.g., IT, finance), greater attention could be directed toward digital well-being and psychological detachment from work. Tailoring interventions to sector-specific stressors enhances both their effectiveness and practical relevance.

Third, organizational managers can play an important role in enhancing employees’ lunch break environment. Providing conducive spaces for breaks, such as designated break rooms or common areas (48), enables employees to recuperate from daily workloads and mitigates the adverse effects of residential road traffic noise on mental health and work performance. To operationalize the environmental factors identified in this study, organizations should invest in designing dedicated rest spaces that facilitate recuperation. Such spaces should be equipped with ergonomic facilities (e.g., reclining chairs or nap pods), controllable environmental features (e.g., adjustable air conditioning and dimmable lighting), and designated quiet zones to promote mental disengagement. In addition to these physical interventions, organizational policies should also be implemented. For instance, the introduction of a formally communicated “Right to Disconnect” policy during lunch breaks, which discourages meetings and work-related communications, can help protect the designated break period. Policy support of this kind is considered necessary to ensure that environmental resources exert their intended restorative effects. Importantly, the effectiveness of these lunch break arrangements may vary across cultural and regional contexts. In settings where extended midday breaks are customary (e.g., East Asia, Southern Europe), organizations may institutionalize longer rest periods. By contrast, in contexts with shorter or less formalized breaks (e.g., Northern Europe, North America), micro-breaks, quiet zones, or flexible scheduling may be more practical.

### 5.3 Research limitations and prospects

The primary limitations of this study relate to several aspects. First, regarding external validity, the representativeness of the sample could be improved. Although the study included urban employees from nearly all provinces in mainland China, approximately 30% of respondents were concentrated in Beijing, which may limit the geographical generalizability of the findings. Furthermore, as data were collected via an online platform, the sampling frame primarily captured individuals with digital access and literacy. While this method was suitable for reaching a national sample of urban employees, who generally exhibit high internet penetration, it may underrepresent groups with limited online connectivity. To strengthen the validity and generalizability of future findings, subsequent research should broaden the geographical scope to achieve a distribution more closely aligned with China’s provincial composition and adopt mixed-mode data collection methods (such as supplementing online surveys with community-based or paper-based approaches) to capture participants from more diverse digital backgrounds.

Second, regarding measurement validity, although this study sought to mitigate participants’ concerns through anonymous questionnaires, reliance on self-reports may still introduce bias, particularly for sensitive constructs such as workplace aggressive behaviors. These measures are vulnerable to social desirability and self-presentation effects, which can result in under-reporting or distortion of behaviors. Future research could address this limitation by complementing self-reports with peer or supervisor ratings, behavioral observations, or organizational records (e.g., absenteeism, disciplinary data). To strengthen the ecological validity of noise exposure assessment, future studies might also integrate subjective reports with objective indicators (e.g., portable decibel meters). Drawing on such multiple sources may help reduce common method variance, improve measurement robustness, and yield more credible evaluations of workplace negative behaviors.

Third, this study examined the influence of RRTNA on employees’ work withdrawal and workplace aggressive behaviors, incorporating mental health complaints as a mediating factor. The results show that mental health complaints partially mediate the relationship between RRTNA and workplace aggressive behaviors, suggesting the presence of additional mediators. Future research should investigate other potential mechanisms, such as low life satisfaction (68), to develop a more comprehensive understanding of how these constructs are linked.

Fourth, this study investigated the organizational lunch break environment as a contextual moderator, emphasizing its cultural significance in China. While this focus offers a novel, contextually grounded contribution, other organizational factors, such as leadership quality (69), could also serve to buffer the negative impact of RRTNA. Future research should further explore these moderators to develop a more comprehensive understanding of how organizational systems may help mitigate the cross-domain spillover of environmental stressors from home to work.

Fifth, this study primarily investigates the mechanism through which RRTNA influences employees’ negative workplace behaviors, focusing on outcomes. Future research could examine antecedents and boundary conditions of RRTNA. Objective noise levels provide important context, as higher decibel levels generally increase the likelihood of residents perceiving noise annoyance. Building characteristics, including ventilation patterns and window configurations, also shape the indoor noise environment. For instance, mechanical ventilation and noise-reducing glass can attenuate street noise, thereby reducing perceived disturbance. Beyond environmental factors, individual differences are critical. Noise sensitivity, a personality-related trait, directly influences perception and evaluation of noise (37). Broader personality traits, such as the Big Five, may act as antecedents of RRTNA and moderate of psychological and behavioral responses. Incorporating these variables in future research would provide a more nuanced understanding of population heterogeneity and strengthen the theoretical foundations for addressing residential road traffic noise annoyance. Moreover, urban and architectural mitigation strategies warrant greater attention. Urban planning interventions, such as noise barriers, green buffers, and traffic management, can reduce community-level noise exposure. Similarly, building design strategies, including facade optimization and soundproofing materials, can mitigate indoor noise disturbances. Future studies integrating these physical mitigation strategies with individual differences and organizational factors may yield a comprehensive, transdisciplinary framework for addressing the complex challenges of residential road traffic noise.

Last, although this study adopts a quantitative survey design allowing for generalization, it lacks qualitative perspectives on employees’ lived experiences, emotions, and coping strategies in response to residential road traffic noise. Qualitative insights could illuminate how employees perceive and manage noise disturbances, and how these processes influence workplace behaviors. Prior research indicates that individuals may use emotion-focused coping (e.g., mindfulness) (70) or cognitive reframing (71). Such nuanced experiences are often difficult to capture through standardized surveys. Therefore, future research should employ qualitative or mixed-method approaches, such as interviews or diary studies, to complement quantitative data and provide a more contextualized understanding of employees’ responses to RRTNA.

## Data Availability

The data that support the findings of this study are not publicly available due to restrictions imposed by the research team’s data sharing policy. However, data may be made available upon reasonable request and subject to approval by the team. Requests to access the datasets should be directed to Yufei, yff667@outlook.com.
